# Synthesis of N-A-S-H Gels via Sol–Gel and Hydrothermal Methods: Composition, Microstructure, and Dynamic Adsorption Characteristics for Sr^2+^

**DOI:** 10.3390/ma19194035

**Published:** 2026-09-22

**Authors:** Zhao Zheng, Xinpeng Ye, Jun Yang, Long Shi, Xue Ma, Luping Tang

**Affiliations:** 1 School of Civil Engineering and Architecture, Southwest University of Science and Technology, Mianyang 621010, China; 2 Shock and Vibration of Engineering Materials and Structures Key Laboratory of Sichuan Province, Southwest University of Science and Technology, Mianyang 621010, China; 3 School of Materials and Chemistry, Southwest University of Science and Technology, Mianyang 621010, China; 4 Division of Building Technology, Chalmers University of Technology, 41296 Gothenburg, Sweden

**Keywords:** sol–gel synthesis, hydrothermal synthesis, N-A-S-H gels, Si/Al, dynamic adsorption capacity

## Abstract

Fly ash-based geopolymers are widely used as solidification matrices for intermediate- and low-level radioactive waste liquids. Their performance relies heavily on the adsorption capacity of the primary component, N-A-S-H gels, which directly governs the long-term stability of the solidified waste form. This study prepared N-A-S-H gels using both the sol–gel synthesis and the hydrothermal synthesis method. The effects of the Si/Al ratio, alkalinity, and NaNO_3_ on the structure of N-A-S-H gels were analyzed using EDS, FT-IR, and XRD. Additionally, the dynamic adsorption capacity of N-A-S-H gels for Sr^2+^ under varying solution temperatures and alkaline conditions was evaluated and fitted using the Thomas and Yoon–Nelson models. For sol–gel-synthesized gels, a lower initial Si/Al ratio led to a higher theoretical maximum Sr^2+^ adsorption capacity of 121.71 mg/g at room temperature and neutral pH. For hydrothermally synthesized gels from raw fly ash, the high initial alkalinity reduced the Si/Al ratio but adversely decreased Sr^2+^ adsorption capacity. Raising the temperature to 60 °C improved Sr^2+^ uptake for all gel samples, with a maximum increase of 28.75 mg/g. Under alkaline conditions, Sr^2+^ uptake for all gels decreased significantly, with a minimum theoretical reduction of 31.55 mg/g. The findings of this study provide a theoretical foundation for understanding the radionuclide migration mechanisms of fly ash-based geopolymer solidification forms in complex geological environments.

## 1. Introduction

Large amounts of radioactive liquid waste are generated during nuclear reactor operation, spent fuel reprocessing, and decommissioning of nuclear facilities. Low- and intermediate-level radioactive waste liquids account for over 97% of the total volume, far exceeding that of high-level waste [1]. These wastes contain large amounts of soluble sodium salts together with radionuclides, such as ^137^Cs and ^90^Sr [2]. Due to the zeolitic phases and N-A-S-H gels in geopolymers possessing large specific surface areas and cage-like structures, geopolymers exhibit excellent adsorption effects on radioactive metal ions and heavy metal ions [3]. Thus, geopolymers are considered a highly promising solidification material for low- and intermediate-level radioactive waste instead of cement-based materials [4,5]. Numerous scholars worldwide have conducted research on the adsorption of radioactive ions and heavy metal ions by geopolymers.

He et al. [6] found that chabazite and Na-P1 zeolite formed in fly ash-based geopolymers exhibit excellent static adsorption capacity for radionuclides Sr/Cs. Yang et al. [7] prepared fly ash-based geopolymers via hydrothermal synthesis to study their static adsorption performance for Sr and discovered that NaNO_3_ (the main salt in low- and intermediate-level radioactive waste liquids) reduced the compactness of the geopolymer structure and decreased its Sr adsorption capacity. The diffusion rates of Cs^+^ and Sr^2+^ in aqueous solutions are highly correlated with the critical pore size of the binder. As the critical pore size of fly ash-based geopolymers is significantly smaller than that of Portland cement, geopolymers obtain superior physical barrier effects in retarding nuclide diffusion. Jang et al. [8] evaluated the leaching behavior of Cs and Sr in geopolymers and found that the diffusion rates of Cs and Sr in fly ash-based geopolymers are 10^3^ and 10^4^ times lower than in Portland cement, which demonstrated the excellent immobilization performance of geopolymers. Zhu et al. [3] discovered that the zeolite structure transformed from N-A-S-H gel under high-temperature conditions exhibits high immobilization efficiency for radionuclide ions. Moreover, the adsorption capacity of N-A-S-H gel and zeolite-like products for radionuclides far exceeds that of C-S-H gel in silicate cement, particularly for monovalent nuclides like Cs^+^. Mukiza et al. [2] noted that N-A-S-H gel maintains a stable structure under irradiation, ensuring the long-term integrity of the bulk solidified form.

Despite these advances, the adsorption performance of pure-phase N-A-S-H gel—a typical geopolymer product—toward radioactive ions remains insufficiently studied. Currently, there are three main methods for preparing N-A-S-H gel. The first is the organic steric solution embedding method [9], where fly ash, sodium hydroxide, and sodium silicate are dissolved in distilled water in specific proportions and mixed with an activating solution containing polyethylene glycol to form a uniform paste, which is then cured to obtain the gel. The second method is the sol–gel method [10], where sodium hydroxide solid is added to a sodium silicate solution with stirring, followed by the addition of aluminum nitrate solid. The third method is hydrothermal synthesis [11], in which nano-silica, alumina, and sodium hydroxide are sequentially added to deionized water, mixed, and then sealed for constant temperature curing.

Although various synthesis routes exist, most rely on pure chemical reagents, with only a few using fly ash as the main raw material. Additionally, the ratios of Si, Al, and Na in the formulation significantly impacted the adsorption performance of N-A-S-H gel. Wang et al. [12] used molecular dynamics simulations to study the structural and mechanical properties of N-A-S-H gel with and without radioactive Sr^2+^ and Cs^+^ and found that N-A-S-H gel effectively immobilizes both radioactive Sr^2+^ and Cs^+^ ions, and they found that the gel effectively immobilizes both ions, with the best effect for Sr^2+^, followed by Cs^+^.

In summary, current research on the Sr adsorption performance of fly ash-based geopolymers primarily focuses on static adsorption properties and molecular simulations of N-A-S-H gel and zeolites within geopolymer matrices. There are limited reports on the dynamic adsorption performance of pure-phase N-A-S-H gel for Sr. Therefore, in this work, we synthesized single-phase N-A-S-H gels via hydrothermal and sol–gel methods using different NaOH and NaNO_3_ contents (the latter simulating the main salts in low- and intermediate-level radioactive waste liquids) and systematically investigated the dynamic adsorption kinetics under coupled temperature and chemical conditions.

## 2. Materials and Experiments

### 2.1. Materials

The fly ash was sourced from Jiangyou Power Plant in Sichuan Province, China. Its oxide composition is presented in Table 1, which shows that silicon and aluminum contents collectively account for over 80% of the total composition, with a Si/Al ratio of 2.31. The D_50_ of fly ash (the particle size when the cumulative percent of pass is 50%) is 17.3 μm. The oxide composition of fly ash is shown in Table 1, and the SEM microstructures and XRD patterns can be seen in reference [7] published by our team.

### 2.2. Sample Preparation and Experiment

#### 2.2.1. Hydrothermal Synthesis Method

According to the proportions in Table 2, fly ash-based N-A-S-H gel was prepared using the hydrothermal synthesis method, with a fixed water volume of 800 mL for all formulations. Firstly, deionized water was measured into a beaker, followed by sequential addition of fly ash and NaOH (flake), and it was then stirred at a constant temperature of 60 ± 3 °C (water bath) at 330 r/min for 8 h. After standing to allow phase separation, the supernatant was collected. For group FA1, anhydrous ethanol was added to the supernatant to completely precipitate flocculent matter. For groups FA2, FA3 and FA4, concentrated nitric acid was added to acidify the supernatant until stable flocculent matter formed. Subsequently, vacuum filtration, washing, and drying were performed to finally obtain fly ash-based N-A-S-H gel. The amount of nitric acid added corresponding to the initial alkalinity is shown in Table 3.

#### 2.2.2. Sol–Gel Synthesis Method

According to the proportions in Table 4, N-A-S-H gels were prepared through the sol–gel synthesis method. Deionized water was used to prepare 0.1 M, 0.2 M, and 0.3 M NaSiO_3_ solutions, 0.1 M Al(NO_3_)_3_ solution, and 10 M NaOH solution. Then, 200 mL of NaSiO_3_ solution and 10 mL of NaOH solution were stirred at 20 °C and 330 rpm for 30 min. Subsequently, 200 mL of Al(NO_3_)_3_ solution was added to the mixture, and the reaction was allowed to proceed for 24 h.

Next, the suspension was centrifuged at 5000 rpm for 5 min to separate the liquid. The precipitate was washed with deionized water by centrifuging at 5000 rpm for 5 min, while this washing process was repeated twice to remove residual NO_3_^−^ ions. Finally, the deionized water was replaced with anhydrous ethanol for one additional wash to remove moisture. After drying, the N-A-S-H gels were obtained.

#### 2.2.3. Dynamic Adsorption Experiment Method

A column (dynamic) dissolution adsorption/desorption experimental platform was established as shown in Figure 1. The N-A-S-H gels prepared by both the hydrothermal and sol–gel methods were used in the adsorption experiments. The prepared N-A-S-H gels were uniformly ground to approximately a 50–80 mesh particle size; 1 g of the samples with a height of 0.2~0.3 cm was placed on the sieve plate at each experiment. Strontium chloride solutions with Sr^2+^ concentrations of 100 mg/L were prepared in 2 L volumetric flasks at 25 °C. Through a peristaltic pump (instrument: LM intelligent filling peristaltic pump, China) at 30 r/min (actual volumetric flow rate: 2.43 mL/min), the solution was pumped into the adsorption column (volume: 12 mL; diameter: 0.5 cm) under neutral pH or pH = 12 at 25 °C or 60 °C. Then, the adsorbed liquid was extracted continuously for 8 h, with 10 mL samples of the adsorbed liquid collected every 20 min. After filtration through 0.45 μm syringe filters, the Sr^2+^ concentration was measured, and the equilibrium adsorption capacity and adsorption efficiency of the N-A-S-H gels for Sr^2+^ were calculated using corresponding formulas. The data are from single measurements.

#### 2.2.4. Characterization Methods

The structural characterization of samples prepared by sol–gel or hydrothermal methods was performed using the following analytical techniques. X-ray diffraction (XRD) analysis was conducted on a SmartLab instrument (Rigaku, Tokyo, Japan) with Cu-Kα radiation at a scanning range of 5°–80° and a speed of 20°/min. Fourier-transform infrared (FTIR) spectra were recorded on an IS5 spectrometer (Thermo Fisher Scientific, Waltham, MA, USA) with KBr-pressed pellets prepared under 12–14 MPa pressure for 15 s from 400 to 4000 cm^−1^. Morphological examination was carried out by scanning electron microscopy (SEM) using a Zeiss Sigma 360 instrument (Carl Zeiss Microscopy GmbH, Jena, Germany) with gold-sputtered bulk samples. Elemental composition was determined by energy-dispersive X-ray spectroscopy (EDS) on an Oxford Ultim 40 system (Oxford Instruments NanoAnalysis, Abingdon, UK) with gold-sputtered bulk samples. The concentration of Sr^2+^ in the outlet solution was detected by an inductively coupled plasma optical emission spectroscopy (iCAP 7000, Thermo Fisher Scientific, USA).

## 3. Results and Discussion

### 3.1. N-A-S-H Gel Synthesized via the Hydrothermal Synthesis Method

#### 3.1.1. Xrd and Ir Analysis

Figure 2 presents the XRD patterns of the N-A-S-H gels prepared from raw fly ash by the hydrothermal synthesis method. FA1, FA2, and FA3 gels all exhibit broad amorphous diffraction peaks with residual SiO_2_ (ICDD#: 97-003-0869). In contrast, the FA4 gel shows distinct diffraction peaks, with a notably intense NaNO_3_ peak (ICDD#: 97-005-6608), attributed to impurity formation during acidification with concentrated nitric acid. Additionally, the amorphous hump near 30° weakened significantly in FA4. This is mainly attributed to the inhibitory effect of NaNO_3_ on N-A-S-H gel formation, as NaNO_3_ crystallizes within the matrix [13]. The presence of NaNO_3_ crystals in the matrix inhibits N-A-S-H gel formation and reduces the purity and polymerization degree of the gel. Furthermore, excessive NaOH was carbonized to Na_2_CO_3_ (ICDD#: 97-006-0311), Na_2_CO_3_·H_2_O (ICDD#: 97-000-1852), and Na_2_CO_3_·7H_2_O (ICDD#: 97-003-5194) during the synthesis process in all the samples [14]. It should be noted that while minor carbonate formation occurs during the synthesis process due to atmospheric CO_2_ exposure, the carbonate content is low and does not significantly affect the dynamic adsorption measurements, as the adsorption experiments were conducted under controlled solution conditions where carbonate interference is minimal.

Figure 3 presents the IR spectra of N-A-S-H gels prepared by the hydrothermal synthesis method. As seen in Figure 3a, because both the preparation and FTIR measurements involved air exposure, and the synthesis was conducted under strongly alkaline conditions, minor carbonate formation occurred via reaction with atmospheric CO_2_. This resulted in characteristic carbonate C-O-C asymmetric stretching peaks (~1590–1390 cm^−1^) [15] in the sample spectra. For the FA4 group, the addition of NaNO_3_ sharpened the N-O infrared spectral peaks [7]. In Figure 3b, the spectral band within 1000–1085 cm^−1^ could be deconvoluted into two components at 1085 and 990 cm^−1^, corresponding to -Si-O-(Si) bridging bonds [16]. The lower wavenumber of the Si-O-T asymmetric stretching peak indicates greater Na^+^ incorporation into the N-A-S-H gel framework, as the substitution of Si^4+^ by Al^3+^ creates negative charges balanced by Na^+^ [17].

The spectra reveal that the decrease in alkalinity through nitric acid addition caused shifts of both Si-O-T asymmetric stretching and Al-O peaks toward higher wavenumbers. This phenomenon is attributed to reduced dissolution of [SiO_4_]^4-^ and decreased Al incorporation at lower alkalinity [16]. For the FA4 group, the additional presence of NaNO_3_ (at concentrations below 200 g/L) may have partially contributed to the transformation of N-A-S-H gel into Na-P1 zeolite [5] and increased the Si-O-T asymmetric peak wavenumbers consequently.

#### 3.1.2. Sem and Eds Analysis

Figure 4 presents the SEM images of N-A-S-H gel prepared by the hydrothermal synthesis method. As shown in Figure 4a, the FA1 gel, prepared through anhydrous ethanol dehydration, exhibits a reticulated porous microstructure with extremely fine particle size. Figure 4b demonstrates that the FA2 gel, prepared by acidification with concentrated nitric acid followed by vacuum filtration and drying, displays a more compact microstructure with similarly flocculent matter. The FA3 gel in Figure 4c, prepared through the same acidification and filtration process, similarly shows a compact morphology with flocculent matter. Figure 4d reveals that the FA4 gel, prepared with NaNO_3_ introduced during acidification, retains the porous network structure but with noticeably larger particle sizes compared to the other three gels. Among them, the FA2 and FA3 gels show the most compact network skeleton structure with the smallest particle sizes. These results confirm that the supernatant from the hydrothermal synthesis can indeed produce gels, while the observed microstructural variations in N-A-S-H gels are likely attributable to differences in alkalinity during preparation. Alkalinity can promote the formation of N-A-S-H gel in the alkali excitation reaction, but the excessive content of Na^+^ from NaNO_3_ and NaOH inhibited the polymerization of N-A-S-H gels [18,19].

Indeed, the N-A-S-H gel particles prepared by ethanol precipitation were excessively fine, which led to dissolution in water and penetration through the filter membrane at the bottom of the adsorption column. Consequently, the FA1 group was excluded from subsequent experiments, while all other groups of N-A-S-H gel powders were maintained with particle sizes between 50 and 80 mesh.

Figure 5 displays the EDS mapping of N-A-S-H gels synthesized via the hydrothermal method, where panels (a), (b), and (c) correspond to FA2, FA3, and FA4 gels, respectively. The average Si/Al ratios are 1.85 for FA2, Na/Al = 1.90 and 1.33 for FA3, and Na/Al = 1.21 and 1.41 for FA4 and Na/Al = 0.75, respectively. These results indicate that lower Si/Al ratios (FA2 > FA4 > FA3) correlate with smaller particle sizes and more compact structures, consistent with the SEM observations in Figure 4. Figure 6 presents the EDS point analysis of these gels. The left side of each panel shows the scanned surface and measurement points, while the right side displays corresponding Si/Al and Na/Al ratios. Figure 6a,c show a linear relationship between the Si/Al and Na/Al ratios in FA2 and FA4. In FA3 (Figure 6b), the Si/Al ratios are approximately 1.82, while the Na/Al ratios are about 1.50. This indicates that the sample with a high content of alkali prefers to form the N-A-S-H gel with the unified composition, but the introduction of NaNO_3_ would break this balance and uniformity. The EDS mapping also reveals that both NaOH and NaNO_3_ reduced the Si/Al and Na/Al ratios of N-A-S-H gels. The activity of mullite was stimulated by the high alkalinity, which enabled more Al elements to participate in the formation of N-A-S-H gel [20,21].

### 3.2. N-A-S-H Gel Synthesized via the Sol–Gel Synthesis Method

#### 3.2.1. Xrd and Ir Analysis

Figure 7 presents the XRD patterns of N-A-S-H gels prepared by the sol–gel synthesis method from raw fly ash. All three gels exhibit a single amorphous diffraction peak. The diffraction peaks for N1, N2, and N3 are located at 29.87°, 29.12°, and 28.48°, respectively, following the order N1 > N2 > N3. The FTIR spectra in Figure 8 also confirm the formation of N-A-S-H gels. Combined with the particle sizes from Figure 9 (N1 having the smallest particle size) and the Si/Al ratios from Figure 10 (N3 > N2 > N1), it demonstrates that as the Si/Al ratio decreases, the particle size of N-A-S-H gels decreases while the diffraction peaks shift to higher 2θ angles. This finding is consistent with the structure predicted by molecular dynamics simulations reported by Chen et al. [22].

Figure 8 displays the IR spectra of N-A-S-H gel prepared by the sol–gel synthesis method. As shown in Figure 8a, due to air exposure during both preparation and IR testing along with the strongly alkaline synthesis conditions, minor carbonate formation occurred from reaction with atmospheric CO_2_, which resulted in the characteristic carbonate C-O-C asymmetric stretching peaks (~1590–1390 cm^−1^) in the spectra [15]. Figure 8b reveals that as the Si/Al ratio increases, the Si-O-T asymmetric stretching band shifts slightly toward higher wavenumbers, corresponding to higher vibrational energy. This phenomenon is related to the fact that Si-O-Si bonds vibrate at higher wavenumbers than Si-O-Al bonds [10]. Because Al-O bonds are longer and have smaller force constants than Si-O bonds, the force constant of Si-O-Al is smaller than that of Si-O-Si [23]. However, the constant alkali concentration in the system prevented a significant increase in depolymerized [SiO_4_]^4−^ concentration, while the fixed Al concentration also limited gel formation. Notably, the hydrothermally synthesized fly ash-derived gels and these sol–gel-synthesized N-A-S-H gels with different Si/Al ratios exhibit remarkably similar structures, and all of them show sharp Si-O-T asymmetric stretching peaks within the 997–1003 cm^−1^ range [16].

#### 3.2.2. Sem and Eds Analysis

Figure 9 shows SEM images of the sol–gel-synthesized N-A-S-H gels. As shown in Figure 9a, the N1 gel obtained through vacuum filtration and drying exhibits a reticulated porous microstructure. Figure 9b demonstrates that the N2 gel displays a similar porous network morphology but with additional flocculent deposits on its surface. The N3 gel in Figure 9c shows comparable porous network characteristics, also with surface flocculent deposits. Among these, the N1 gel has the most compact network skeleton and finer particles, while the N3 gel shows the highest degree of polymerization. These results confirm that the sol–gel process can effectively produce N-A-S-H gels, and that the microstructural variations are likely attributable to differences in the Si/Al ratio. A higher Si content promotes N-A-S-H gel formation within the matrix [24].

Additionally, the SEM images (Figure 4 and Figure 9) show that the gel microstructures are generally uniform within each sample group, with consistent particle size and network morphology across different regions of the same sample. These observations collectively confirm that both synthesis methods yield reasonably homogeneous N-A-S-H gels.

Figure 10 shows the EDS mapping of N-A-S-H gels prepared by sol–gel synthesis, where panels (a), (b), and (c) correspond to N1, N2, and N3 gels, respectively. The measured Si/Al ratios were 1.13 (Na/Al = 0.82) for N1 gel with an initial Si/Al setting of 1, 1.28 (Na/Al = 1.04) for N2 gel with an initial Si/Al = 2, and 1.39 (Na/Al = 1.03) for N3 gel with an initial Si/Al = 3, respectively. The final Si/Al ratios followed the order N3 > N2 > N1. Combined with Figure 9, these results demonstrate that lower Si/Al ratios yield N-A-S-H gels with smaller particle sizes and more compact structures [25], which is consistent with the conclusions drawn in Section 3.1.2.

Figure 11 shows the EDS point analysis for the sol–gel samples, where panels (a), (b), and (c) correspond to N1, N2, and N3 gels, respectively. The left side of each panel shows the scanned area and measurement points, while the right side shows the corresponding Si/Al and Na/Al ratios. Figure 11a–c demonstrate that all Si/Al ratios fall within a relatively narrow range, and the average Si/Al ratios follow the trend of the initial ratios. Although the Na/Al ratios are relatively scattered, higher Na/Al ratios are obtained at higher Si/Al ratios. This indicates that the high initial Si/Al ratios contributed to the formation of N-A-S-H gels with high Si/Al and Na/Al ratios [20,25], and the sol–gel method produces compositionally homogeneous N-A-S-H gels, which agrees with the conclusion of the EDS mapping in Figure 10.

### 3.3. Dynamic Adsorption Properties of N-A-S-H Gel

#### 3.3.1. Model of Dynamic Adsorption

The breakthrough curve demonstrates the dynamic adsorption performance of the adsorbent when continuously fed liquid comes into contact with it in practical engineering applications. Exploring the parameters of the breakthrough curve is of great significance for elucidating the operational performance. The breakthrough behavior of the N-A-S-H gels was evaluated in the context of practical wastewater treatment.

The breakthrough time t_b_ refers to the time required for the effluent concentration below the filter cartridge to reach 0.1% of the initial solution concentration, while the saturation time t_s_ refers to the time required for the effluent concentration below the filter cartridge to reach 95% of the initial concentration. For a given bed depth, the breakthrough curve represents the integral of the Sr^2+^ concentration over time.

The equilibrium adsorption capacity (q_e_, mg/g) was calculated using Equation (1) [26,27]:

(1) $$q_{e}=\frac{Q}{1000m}\int_{t=0}^{t=total}(C_{0}-C_{t})dt$$

where m is the dry weight of the adsorbent (g), Q is the flow rate (mL/min), C_0_ is the initial Sr^2+^ concentration (mg/L), and C_t_ is the effluent concentration at time t (mg/L).

The total mass of Sr^2+^ entering the column (mt, g) and the length of the mass transfer zone (MTZ, cm) are calculated using Equations (2) and (3), respectively:

(2) $$m_{t}=\frac{C_{0}Q}{1000}\int_{t=0}^{t=total}\left(C_{0}-C_{t}\right)dt$$

(3) $$MTZ=Z\left(1-\frac{t_{b}}{t_{s}}\right)$$

To elucidate the dynamic adsorption mechanism of N-A-S-H gel for Sr^2+^, the Thomas model [28] (Equation (4)) and the Yoon–Nelson model [29] (Equation (6)) were applied to fit the dynamic adsorption data and calculate the relevant parameters:

(4) $$\frac{C}{C_{0}}=\frac{1}{1+\exp\left[\left(\frac{K_{TH}}{Q}\right)\left(q_{\max}M-C_{0}Qt\right)\right]}$$

This is simplified as

(5) $$\ln(\frac{C_{0}}{C}-1)=-K_{\mathrm{TH}}C_{0}t+\frac{K_{\mathrm{TH}}q_{\max}M}{Q}$$

where C_0_ is the inlet Sr^2+^ concentration (mg/L), C is the outlet Sr^2+^ concentration (mg/L), K_TH_ is the Thomas rate constant (L/(mg·h)) and reflects the adsorption rate (a higher value indicates faster adsorption), q_max_ is the theoretical saturated adsorption capacity of the adsorbent (mg/g), M is the mass of the adsorbent (g), Q is the fluid flow rate (L/h), and t is time (h).

By substituting the experimental data into Equation (5) and plotting ln(C_0_/C − 1) on the vertical axis while time (t) on the horizontal axis, a fitted straight line is obtained. The slope = K_TH_C_0_, from which K_TH_ (the Thomas rate constant) can be calculated. The intercept is K_TH_q_max_M/Q, and the adsorption capacity q_max_ can be determined by combining the known M and Q.

(6) $$\frac{C}{C_{0}}=\frac{\exp\left(K_{YN}-\Gamma K_{YN}\right)}{1+\exp\left(K_{YN}t-\Gamma K_{YN}\right)}$$

This is simplified as

(7) $$\ln(\frac{C_{0}}{C}-1)=-K_{YN}t+\Gamma K_{YN}$$

where C_0_ is the inlet Sr^2+^ concentration (mg/L), C is the outlet Sr^2+^ concentration (mg/L), K_YN_ is the Yoon–Nelson rate constant (h^−1^)), reflecting the adsorption rate (a higher value indicates faster breakthrough), Γ is the 50% breakthrough time (the time when C/C_0_ = 0.5), and t is time (h).

By substituting the experimental data into Equation (7) and plotting ln (C_0_/C − 1) on the vertical axis with time (t) on the horizontal axis, a fitted straight line is obtained. The slope = −K_YN_, which gives the Yoon–Nelson rate constant. The intercept is ΓK_YN_, and the breakthrough time Γ can be determined by combining the slope.

#### 3.3.2. The Influence of the Si/Al Ratio on the Dynamic Adsorption Behavior of Sr^2+^ by N-A-S-H Gel

Owing to the reduced purity and polymerization degree caused by NaNO_3_ introduction (Figure 2), the FA4 group was excluded from subsequent adsorption experiments. Figure 12 presents the dynamic adsorption experimental results at an inlet Sr^2+^ concentration of 100 mg/L under ambient conditions (25 °C, neutral pH). As shown in Figure 12a, the saturation times at a fixed bed height of 10 mm varied significantly among the gels, following the order N1 gel > N2 gel > N3 gel and FA2 gel > FA3 gel. According to Figure 12b and Table 5, the rate constants (K_TH_) of the Thomas model and (K_YN_) the Yoon–Nelson model were obtained after analyzing the dynamic adsorption data following the order N2 gel > N3 gel > N1 gel and FA2 gel > FA3 gel. The saturated adsorption capacities (q_max_) for Sr^2+^ and the 50% breakthrough times (Γ) followed the descending order: N1 gel > N2 gel > N3 gel and FA2 gel > FA3 gel.

#### 3.3.3. The Influence of Temperature on the Dynamic Adsorption Behavior of Sr^2+^ by N-A-S-H Gel

Figure 13 presents the dynamic adsorption experimental results at an inlet concentration of 100 mg/L, 60 °C, and neutral pH. At 60 °C, the saturation times followed the order N2 gel > N1 gel > N3 gel and FA2 gel > FA3 gel. According to Figure 13 and Table 6, the rate constants (K_TH_) of the Thomas model and (K_YN_) of the Yoon–Nelson model were obtained after analyzing the dynamic adsorption data following the order N3 gel > N2 gel > N1 gel > gel and FA2 gel > FA3 gel. The saturated adsorption capacities (q_max_) for Sr^2+^ and the 50% breakthrough times (Γ) followed the descending order: N2 gel > N1 gel > N3 gel and FA2 gel > FA3 gel. As temperature increases, the mobility of Si and Al atoms is enhanced, and the thermal conductivity increases with higher Si/Al ratios and elevated temperatures [30]. Thus, increasing the temperature from 25 °C to 60 °C enhanced the maximum Sr^2+^ uptake for all gel samples.

**Table 6:** Fitted results of dynamic adsorption experiments for Sr^2+^ at an influent concentration of 100 mg/L under 60 °C under neutral pH.

Samples	K_TH_	q_max_	K_YN_	Γ	R^2^
N1	0.0074 L/(mg·h)	135.56 mg/g	0.74 h^−1^	5.64 h	0.93
N2	0.0092 L/(mg·h)	140.35 mg/g	0.92 h^−1^	5.84 h	0.88
N3	0.0132 L/(mg·h)	111.8 mg/g	1.32 h^−1^	4.65 h	1.00
FA2	0.0104 L/(mg·h)	121.68 mg/g	1.04 h^−1^	4.84 h	0.80
FA3	0.0072 L/(mg·h)	74.33 mg/g	0.72 h^−1^	3.09 h	0.74

#### 3.3.4. The Influence of Alkali on the Dynamic Adsorption Behavior of Sr by N-A-S-H Gel

Figure 14 presents the results at 100 mg/L inlet concentration, 25 °C, and pH = 12. Under alkaline conditions, the saturation times followed the order N1 gel > N2 gel > N3 gel and FA2 gel > FA3 gel. According to Figure 14 and Table 7, the rate constants (K_TH_) of the Thomas model and (K_YN_) the Yoon–Nelson model were obtained after analyzing the dynamic adsorption data following the order N2 gel >N3 gel > N1 gel > gel and FA3 gel > FA2 gel. The saturated adsorption capacities (q_max_) for Sr^2+^ and the 50% breakthrough times (Γ) followed the descending order: N1 gel > N2 gel > N3 gel and FA2 gel > FA3 gel.

**Table 7:** Dynamic adsorption experimental fitting results for Sr^2+^ at 100 mg/L influent concentration under ambient temperature (25 °C) and pH = 12.

Samples	K_TH_	q_max_	K_YN_	Γ	R^2^
N1	0.0274 L/(mg·h)	48.89 mg/g	1.05 h^−1^	5.42 h	0.80
N2	0.0569 L/(mg·h)	30.97 mg/g	2.21 h^−1^	3.38 h	0.87
N3	0.0494 L/(mg·h)	27.68 mg/g	1.92 h^−1^	3.03 h	0.92
FA2	0.0426 L/(mg·h)	21.90 mg/g	1.63 h^−1^	2.39 h	0.94
FA3	0.0439 L/(mg·h)	13.60 mg/g	1.68 h^−1^	1.48 h	0.87

In summary, the Si/Al ratio significantly influences the maximum adsorption capacity of N-A-S-H gels for Sr^2+^. The N1 gel, with the lowest Si/Al ratio among the sol–gel samples, exhibits the highest theoretical maximum adsorption capacity for Sr^2+^. This could be associated with the lower valence state of the aluminum–oxygen tetrahedra than silicon-oxygen tetrahedra; thus, it exhibits stronger adsorption via charge compensation effects [31,32,33]. However, the opposite trend was observed for the hydrothermally synthesized gels. Though the high alkalinity increased the activity of mullite and decreased the Si/Al ratio, more Na^+^ was introduced in the matrices, which inhibited the adsorption capacity of Sr^2+^ [34]. Similarly, the alkaline environment significantly impacts the maximum adsorption capacity of N-A-S-H gels for Sr^2+^. At pH = 12, the maximum adsorption capacity of all samples for Sr^2+^ decreases substantially, which may be attributed to the competitive adsorption of cationic at the presence of abundant Na^+^ [35,36]. Excess Na^+^ occupies the sites originally available for Sr^2+^, thereby reducing the maximum adsorption capacity [37]. However, alkaline conditions greatly accelerated the adsorption process and breakthrough, whereas the temperature effect was relatively modest. The maximum adsorption capacity of all N-A-S-H gel groups for Sr^2+^ also increases at higher temperatures, likely due to the enhanced molecular thermal motion [38]. While our current Thomas and Yoon–Nelson models provide good fits for the breakthrough curves, future work could explore fractional-order models to better capture the complex transport phenomena in N-A-S-H gel adsorbents.

## 4. Conclusions

This study synthesized high-yield N-A-S-H gels through sol–gel synthesis and hydrothermal synthesis methods from raw fly ash. Indeed, the effect of alkalinity and the Si/Al ratio on N-A-S-H gels, as well as the influence of Si/Al ratio, temperature, and alkalinity on the dynamic adsorption performance of Sr^2+^ by N-A-S-H gels, was investigated systematically. The conclusions are as follows:

(1) For the N-A-S-H gels prepared by sol–gel synthesis, increasing the initial Si/Al ratio resulted in a higher final Si/Al ratio. For N-A-S-H gels prepared by hydrothermal synthesis from fly ash, the presence of NaOH and NaNO_3_ decreased the final Si/Al ratios of N-A-S-H gels. Moreover, the purity and the polymerization degree of the N-A-S-H gel decreased due to the introduction of NaNO_3_.

(2) The high content of alkali prefers to form the N-A-S-H gel with the unified composition, but the introduction of NaNO_3_ would break this balance and uniformity. The Si/Al ratio influences the structure of N-A-S-H gels: lower Si/Al ratios led to smaller particle sizes and a more compact gel network.

(3) The dynamic adsorption data can be well fitted with the Thomas and Yoon–Nelson models. As the Si/Al ratio decreased, the theoretical maximum adsorption capacity of N-A-S-H gels for Sr^2+^ increased when the sol–gel chemical synthesis method was used. The maximum theoretical Sr^2+^ adsorption capacity was 121.71 mg/g at room temperature and neutral pH. However, for N-A-S-H gels from the hydrothermal method, the high initial alkalinity reduced the Si/Al ratio but adversely decreased Sr^2+^ adsorption capacity.

(4) Increasing the temperature from 25 °C to 60 °C enhanced the maximum Sr^2+^ uptake for all gel samples. Conversely, an alkaline environment (pH = 12) significantly reduced the adsorption capacity of all gels. Notably, alkaline conditions accelerated the adsorption process and breakthrough to a greater extent, whereas the temperature effect was relatively modest.

## 5. Perspectives

Future work should employ advanced characterization techniques (e.g., solid-state NMR, X-ray absorption spectroscopy) and molecular dynamics simulations to elucidate the exact binding sites and coordination environments of Sr^2+^ within N-A-S-H gel structures at the atomic level. Indeed, the long-term leaching behavior and structural evolution of Sr^2+^-loaded N-A-S-H gels under simulated geological disposal conditions (including temperature cycling, groundwater exposure, and radiation fields) should be systematically evaluated. Moreover, real radioactive waste liquids contain multiple competing cations (Cs^+^, Na^+^, Ca^2+^, etc.). Future studies should investigate the competitive adsorption behavior of Sr^2+^ in multi-component systems to better simulate actual waste treatment conditions.

## Figures and Tables

**Figure 1:**
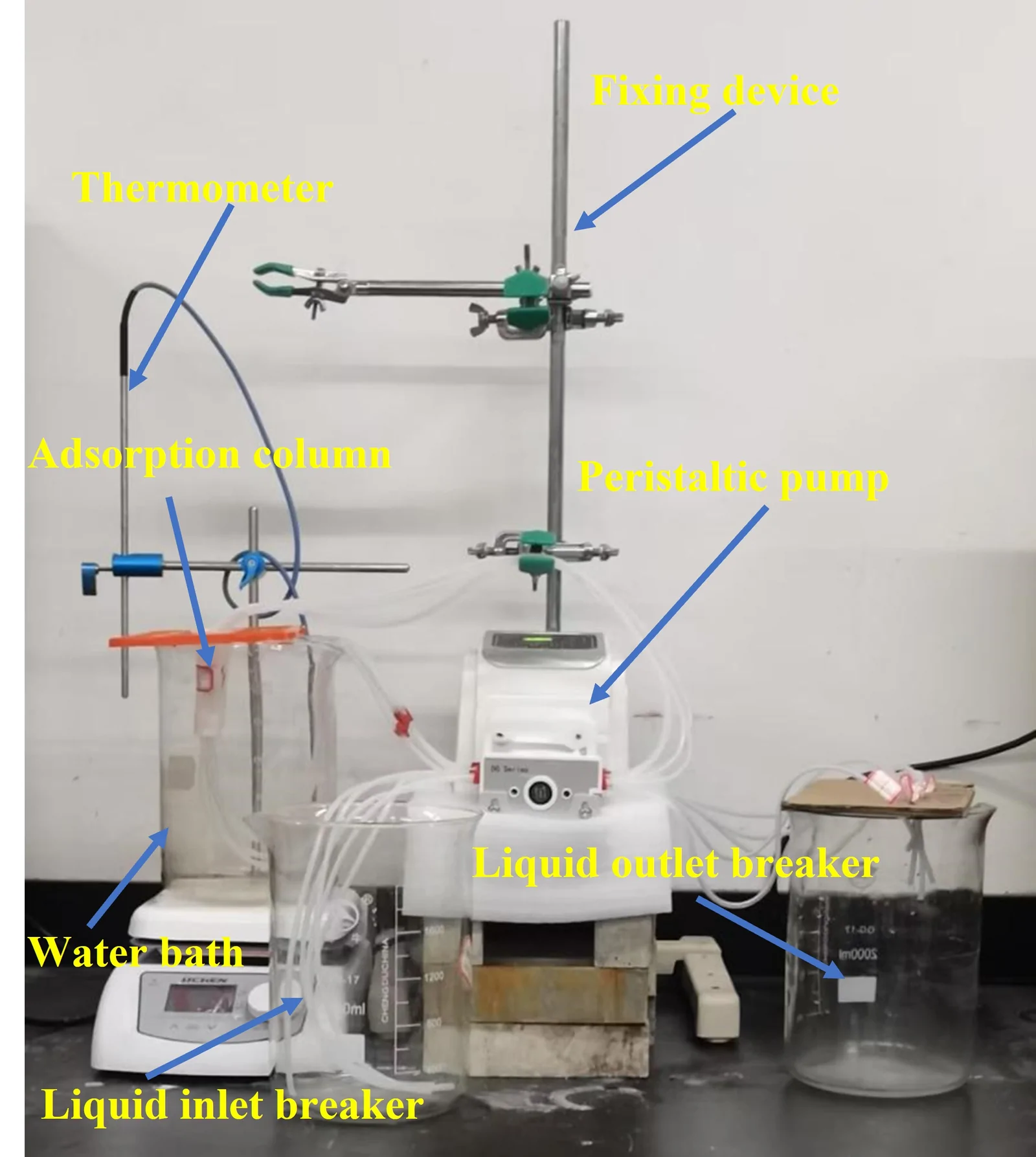
The column (dynamic) dissolution adsorption/desorption experimental platform.

**Figure 2:**
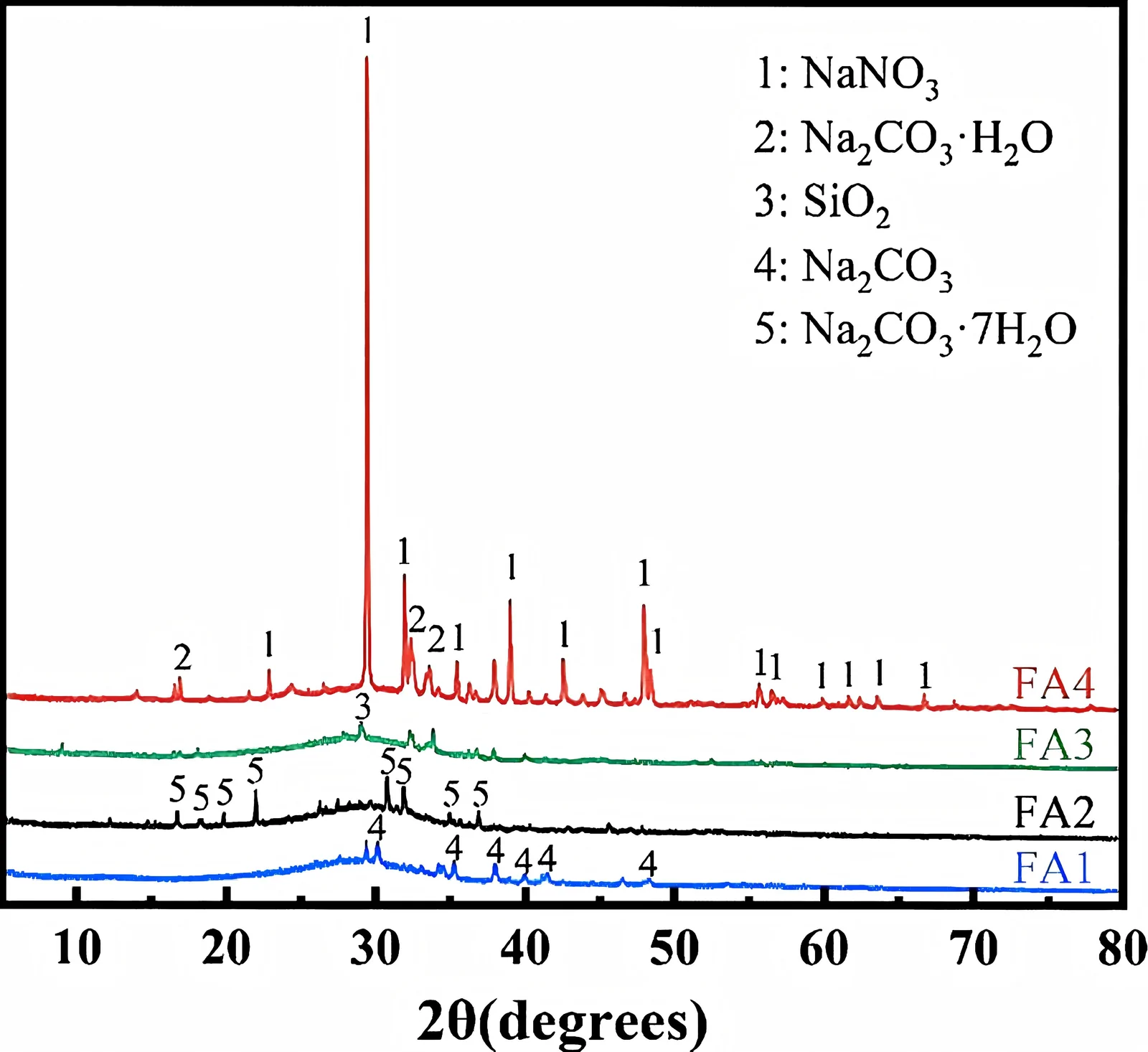
The XRD patterns of N-A-S-H gels synthesized via the hydrothermal method from raw fly ash.

**Figure 3:**
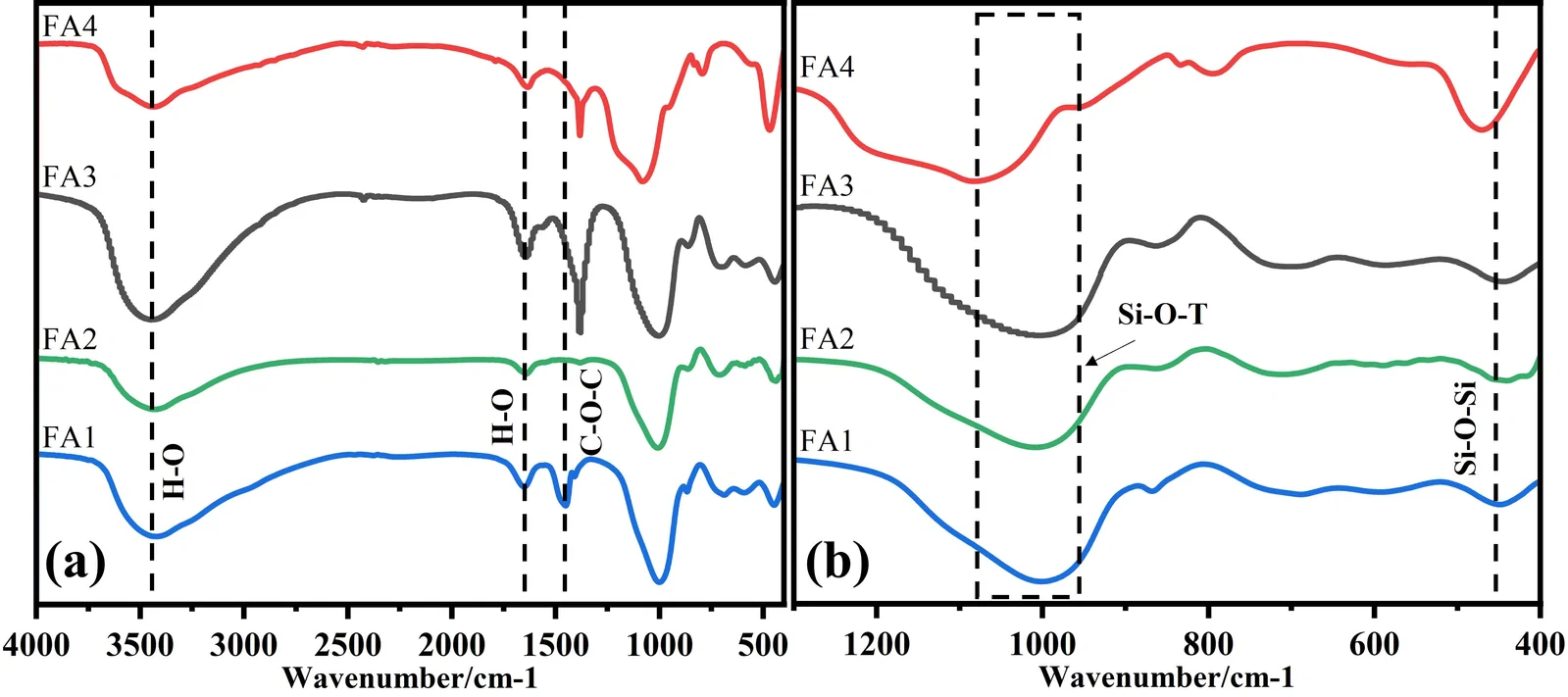
The IR patterns of N-A-S-H gels synthesized via the hydrothermal method from raw fly ash: (**a**) 4000–400 cm^−1^; (**b**) 1300–400 cm^−1^.

**Figure 4:**
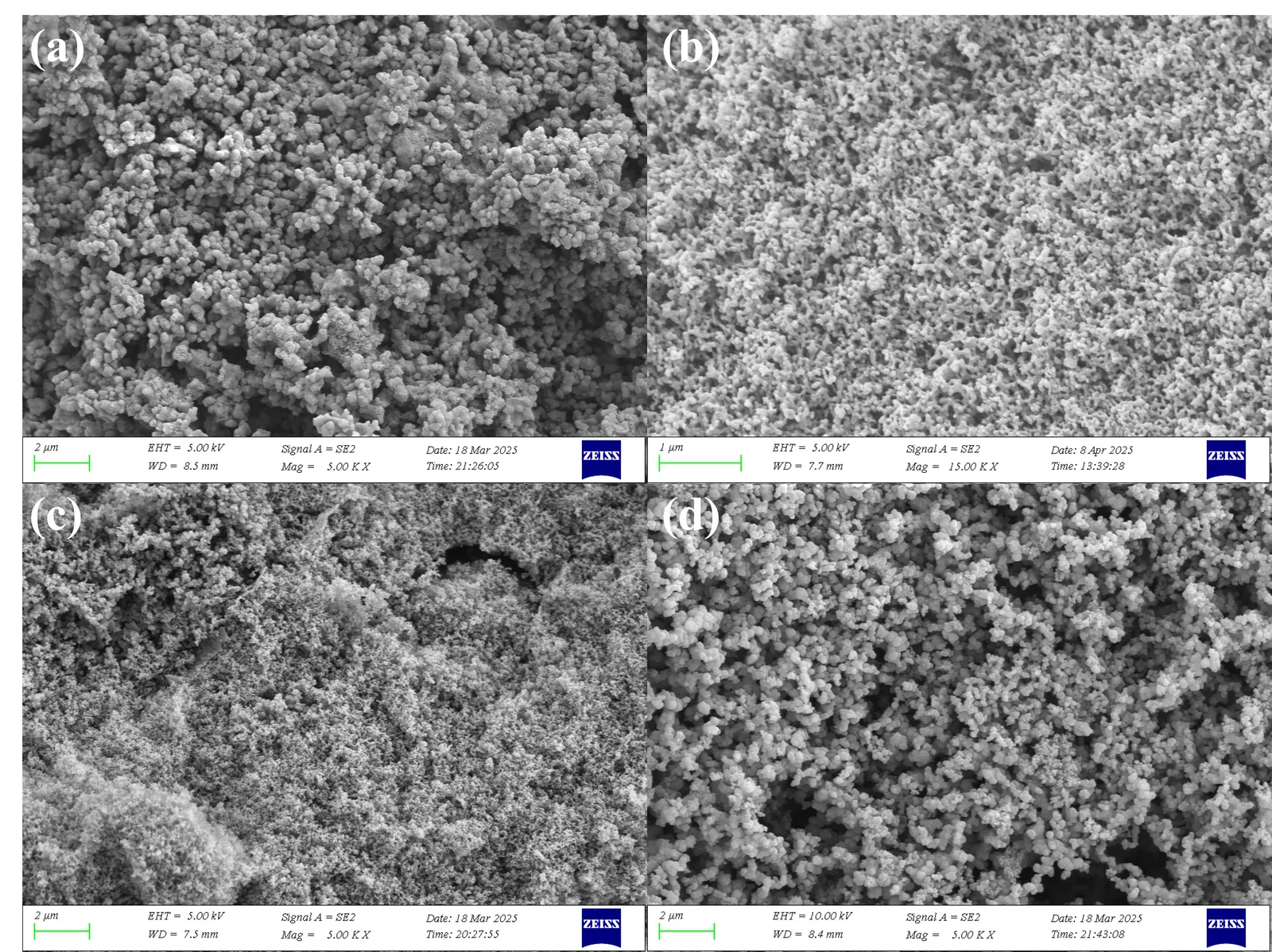
The SEM images of N-A-S-H gels synthesized via the hydrothermal method from raw fly ash: (**a**) FA1; (**b**) FA2; (**c**) FA3; (**d**) FA4.

**Figure 5:**
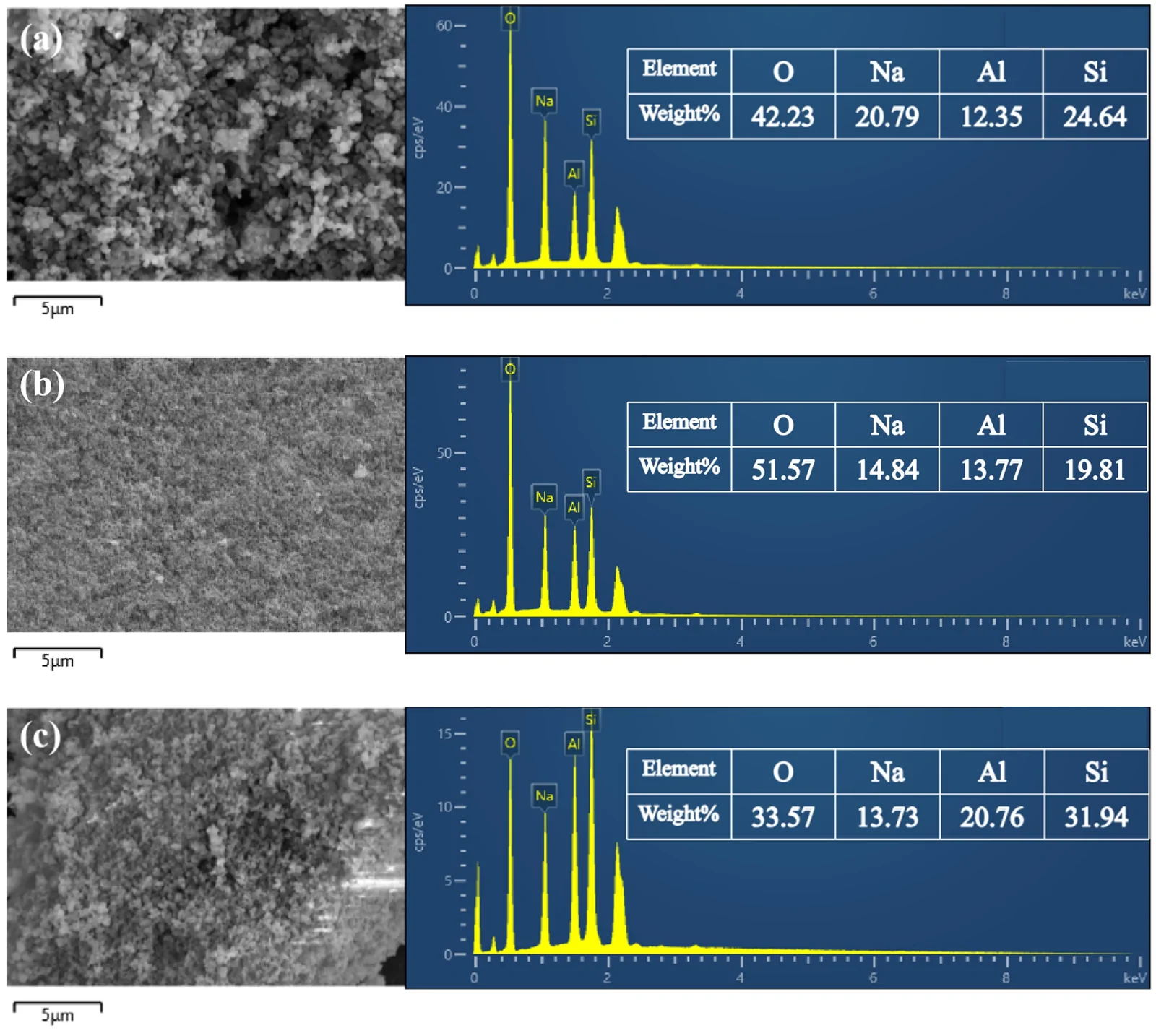
The EDS mapping of N-A-S-H gels synthesized via the hydrothermal method from raw fly ash: (**a**) FA2; (**b**) FA3; (**c**) FA4.

**Figure 6:**
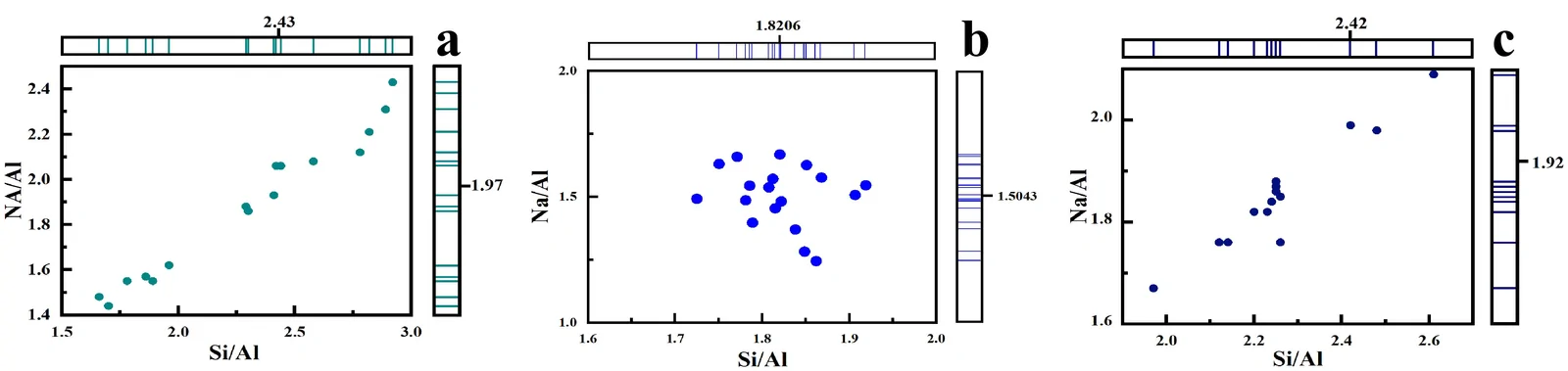
The EDS point of N-A-S-H gels synthesized via the hydrothermal method from raw fly ash: (**a**) FA2; (**b**) FA3; (**c**) FA4.

**Figure 7:**
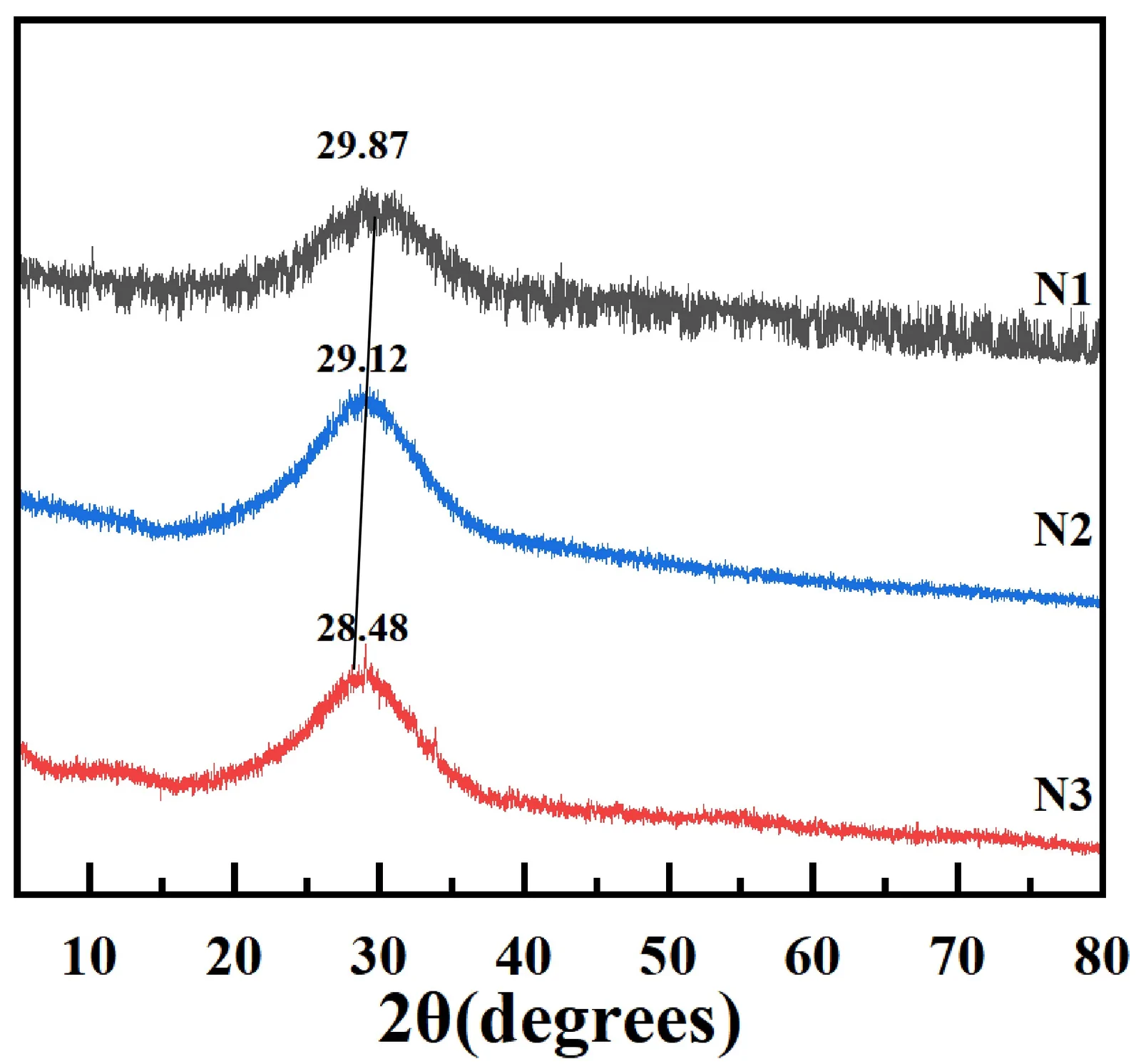
The XRD patterns of N-A-S-H gel by the sol–gel chemical synthesis method.

**Figure 8:**
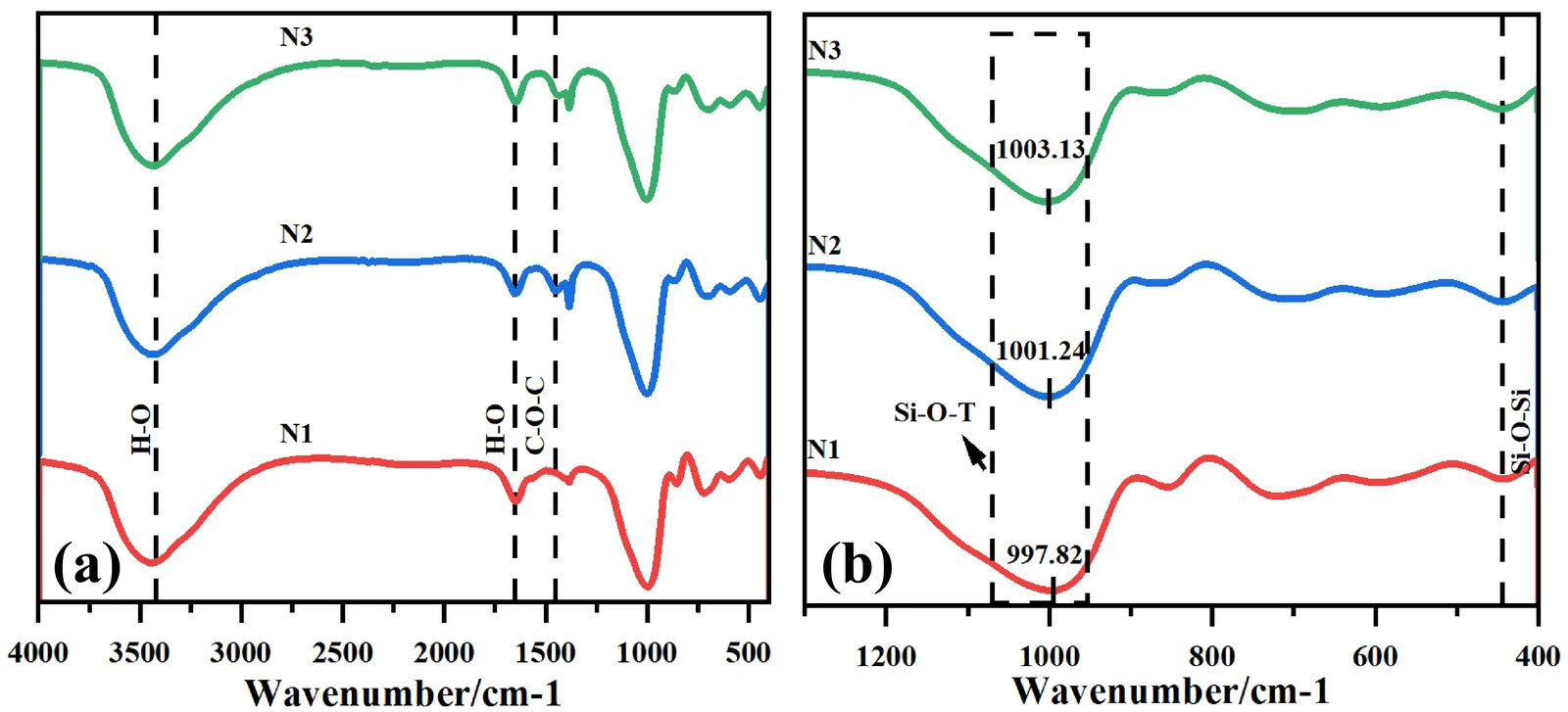
FT-IR spectra of N-A-S-H gel by the sol–gel chemical synthesis method: (**a**) 4000–400 cm^−1^; (**b**) 1300–400 cm^−1^.

**Figure 9:**
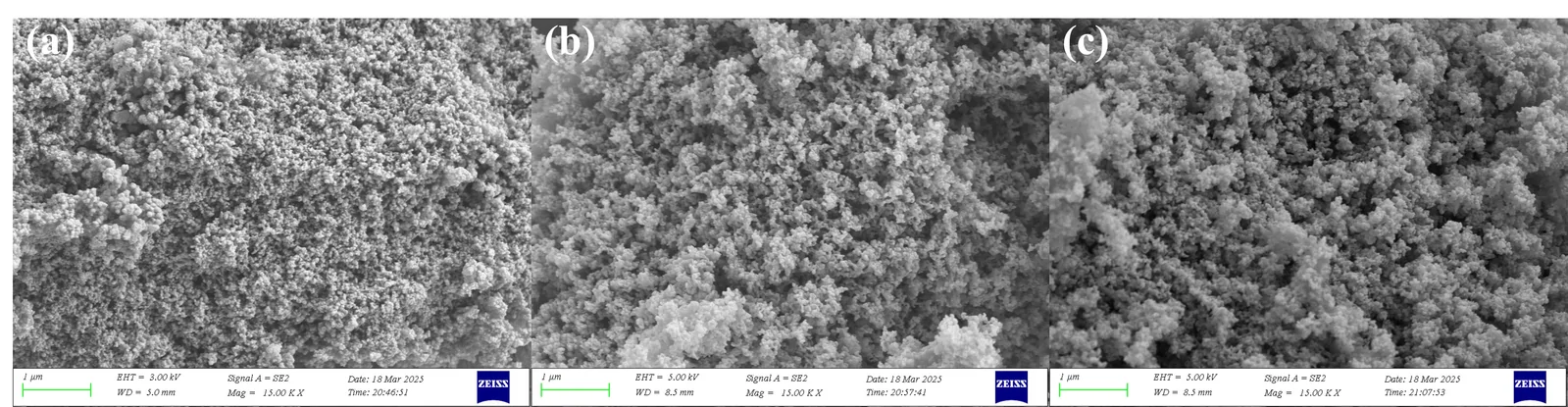
SEM images of N-A-S-H gel by the sol–gel chemical synthesis method: (**a**) N1 gel; (**b**) N2 gel; (**c**) N3 gel.

**Figure 10:**
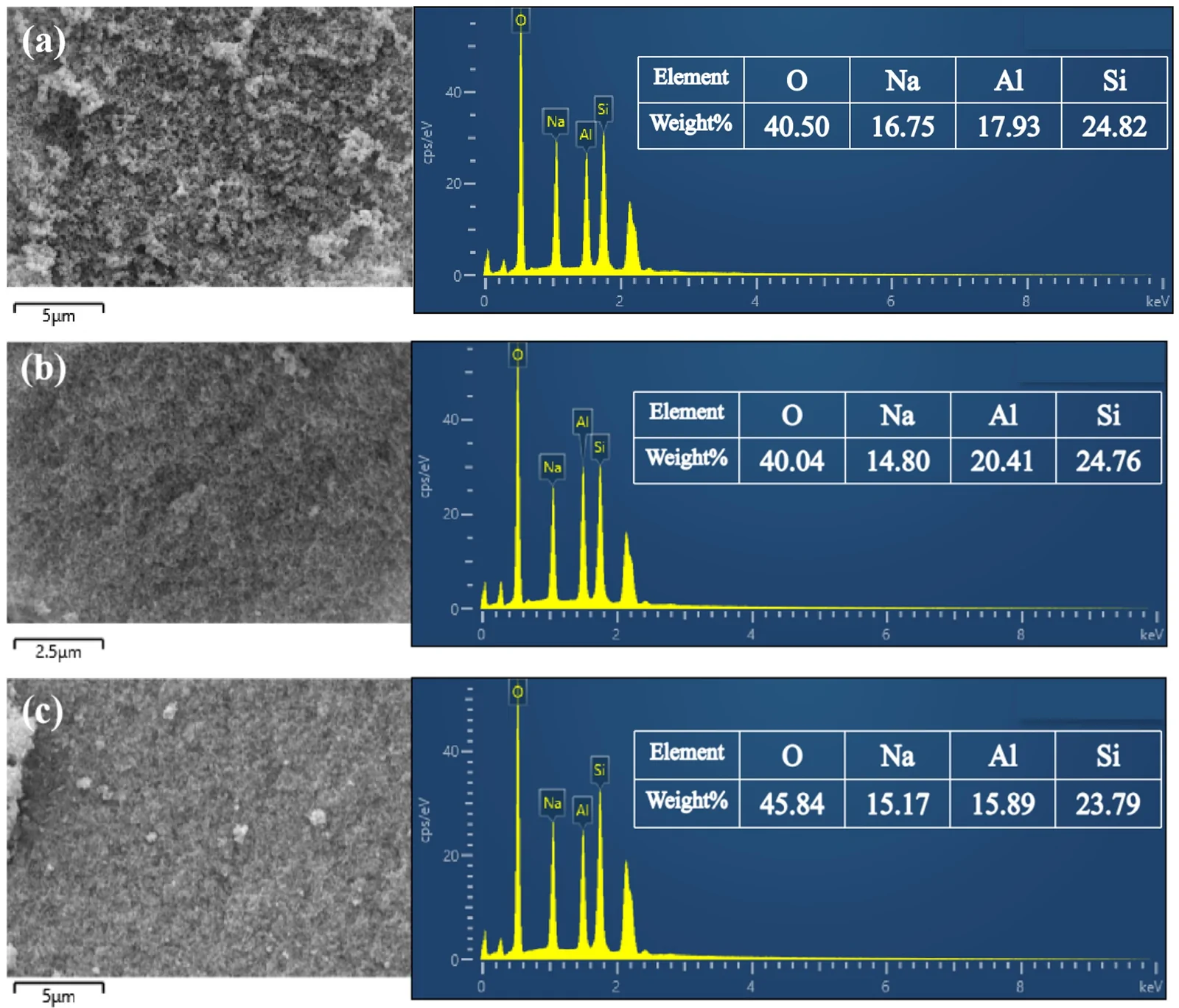
EDS mapping analysis of N-A-S-H gel by the sol–gel chemical synthesis method: (**a**) N1 gel; (**b**) N2 gel; (**c**) N3 gel.

**Figure 11:**
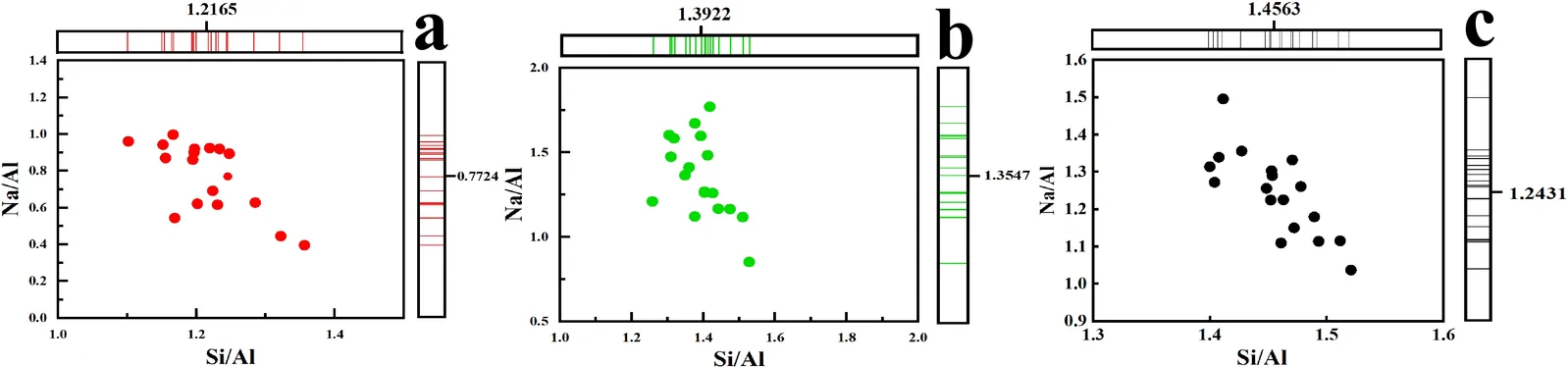
EDS point analysis of N-A-S-H gel by the sol–gel chemical synthesis method: (**a**) N1 gel; (**b**) N2 gel; (**c**) N3 gel.

**Figure 12:**
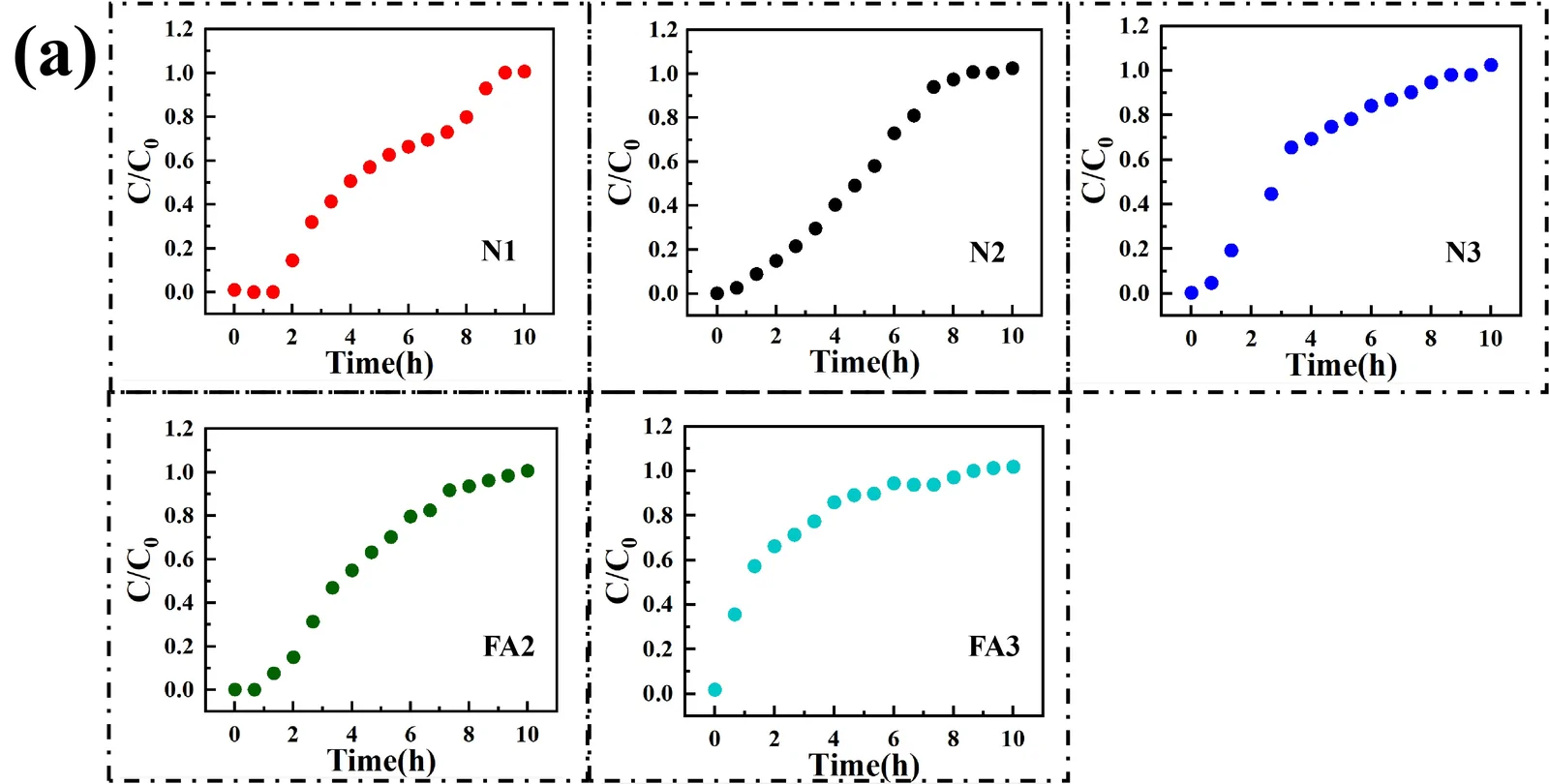

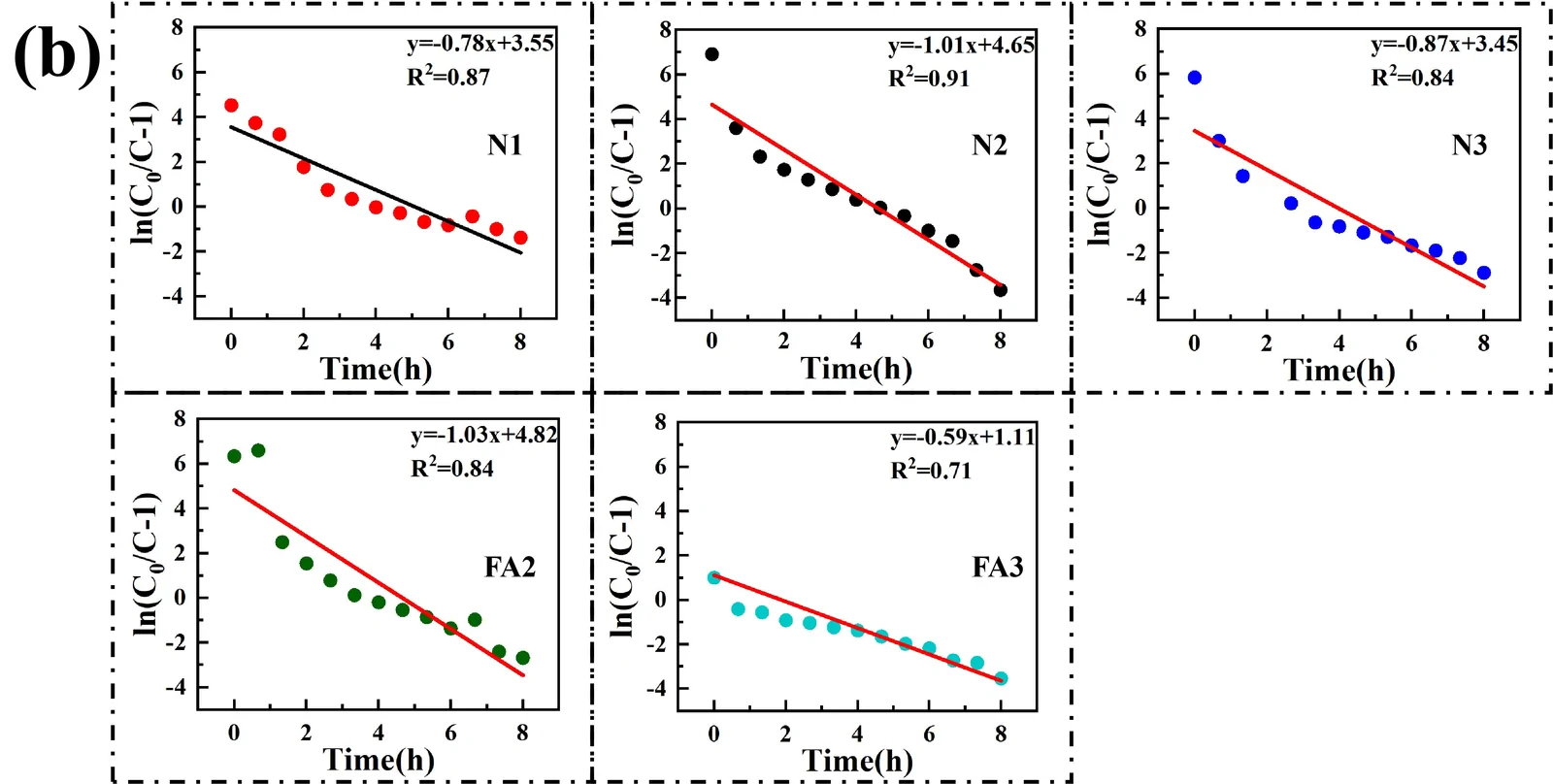
Experimental results of dynamic adsorption for Sr^2+^ at an influent concentration of 100 mg/L under ambient temperature (25 °C) and neutral pH: (**a**) experimental values; (**b**) predicted values by the Thomas model and the Yoon–Nelson model.

**Figure 13:**
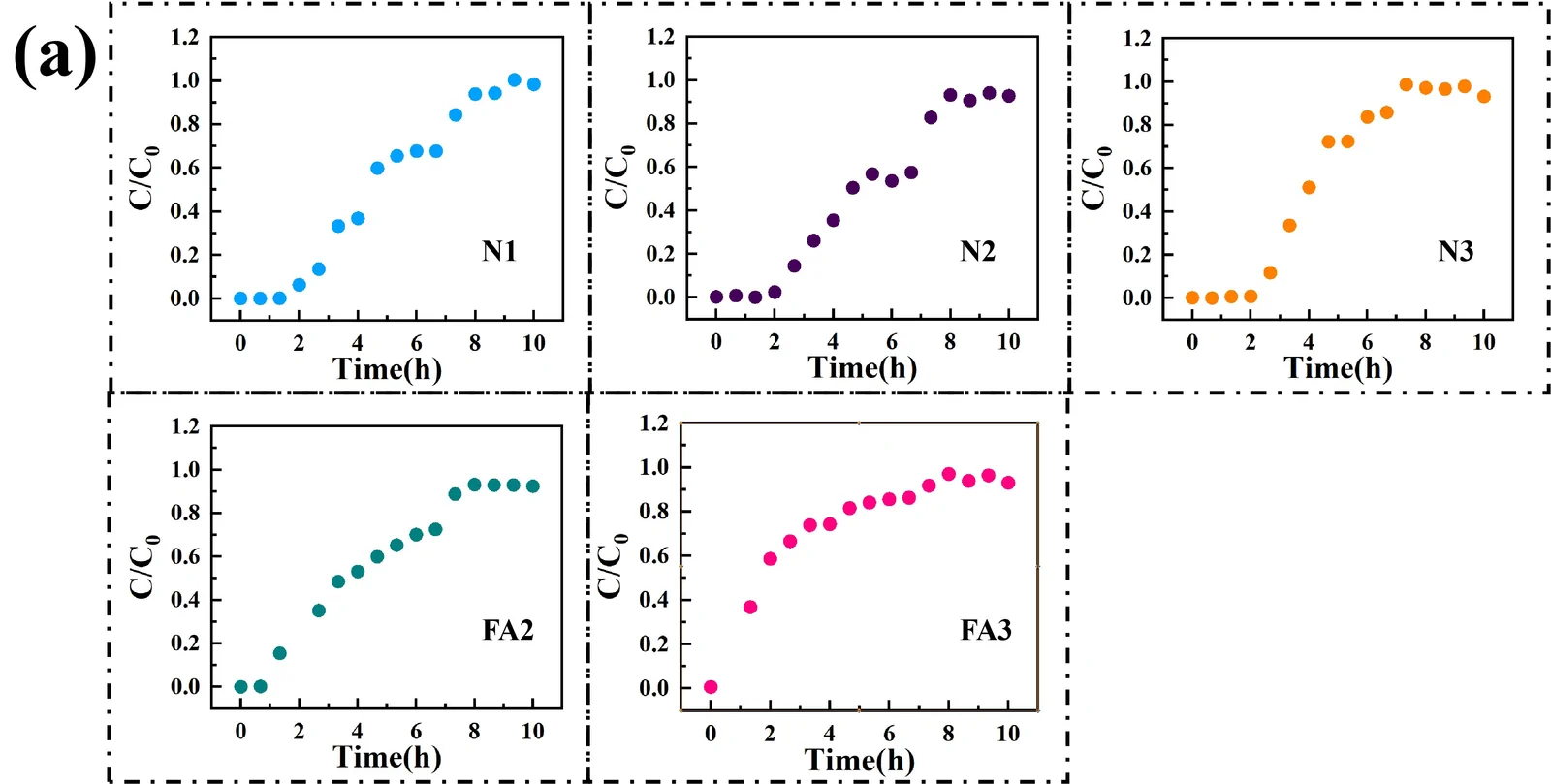

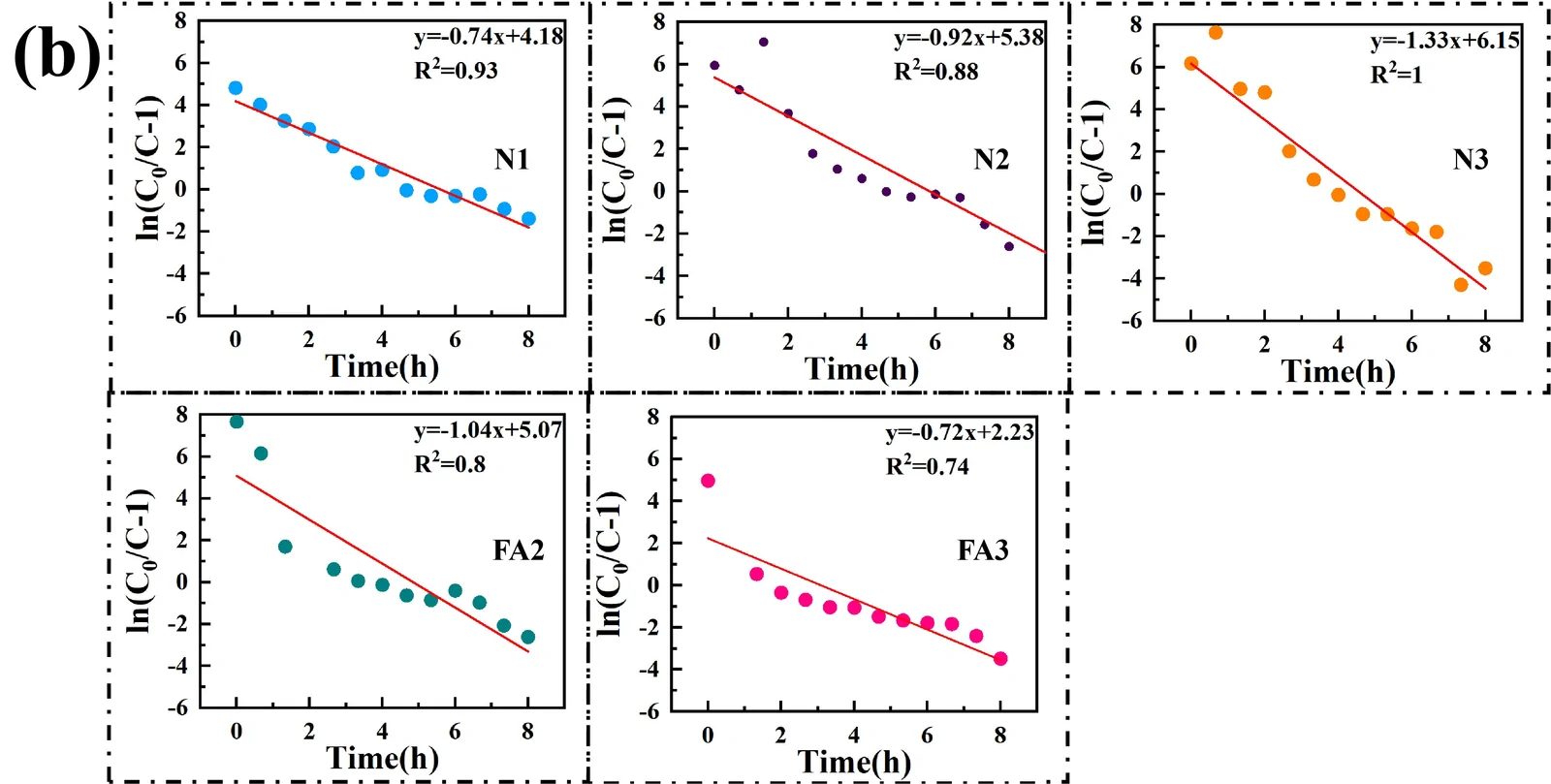
Dynamic adsorption experimental results for Sr^2+^ with an influent concentration of 100 mg/L at 60 °C under neutral pH: (**a**) experimental values; (**b**) predicted values using the Thomas model and the Yoon–Nelson model.

**Figure 14:**
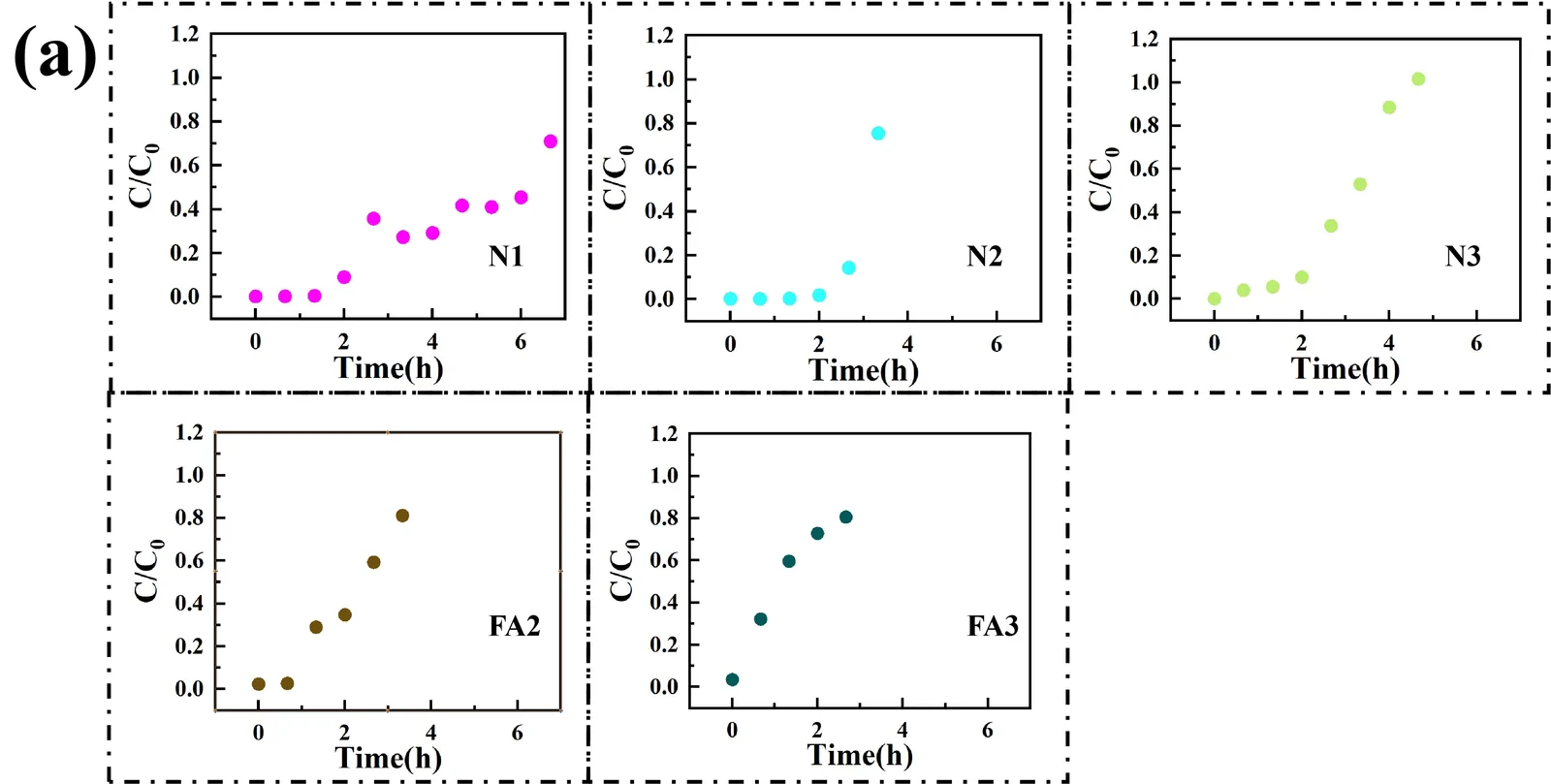

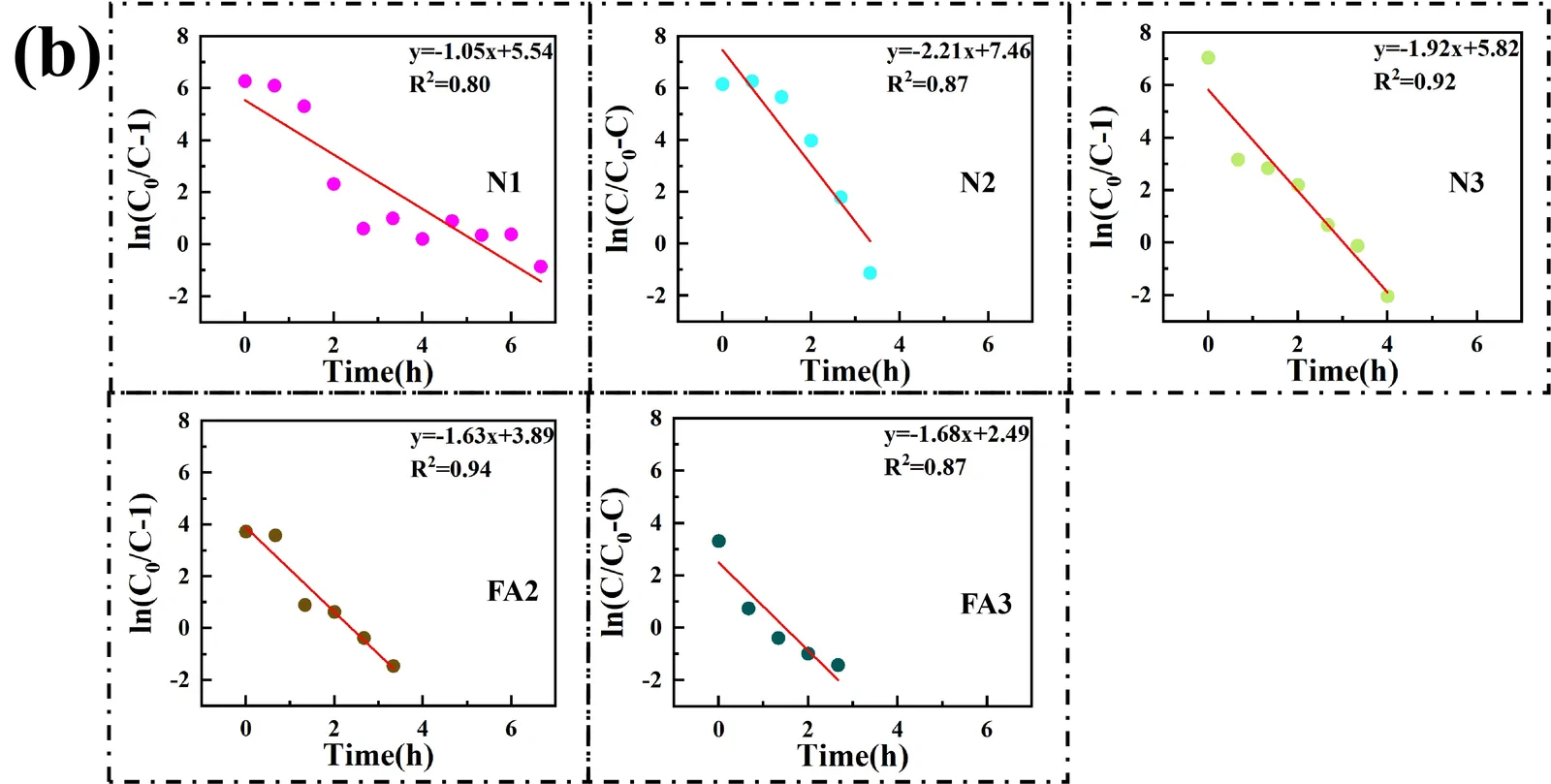
Dynamic adsorption experimental results for Sr^2+^ with an inlet concentration of 100 mg/L under ambient temperature (25 °C) and pH = 12: (**a**) experimental values; (**b**) predicted values using the Thomas model and the Yoon–Nelson model.

**Table 1:** The oxide composition of the fly ash.

Oxide Composition	SiO_2_	Al_2_O_3_	CaO	Fe_2_O_3_	K_2_O	MgO	SO_3_	Others
Content (wt.%)	59.79	21.99	5.55	5.13	3.21	1.35	0.54	2.44

**Table 2:** The formulations of fly ash-based N-A-S-H gel with different alkalinity levels.

Samples	Fly Ash (g)	NaOH (M)	NaNO_3_ (g/L)	Deionized Water (L)	Precipitate Method
FA1	125	4	0	1	anhydrous ethanol
FA2	125	2	0	1	nitric acid
FA3	125	4	0	1	nitric acid
FA4	125	4	100	1	nitric acid

**Table 3:** The volume of concentrated nitric acid added per 50 mL of supernatant.

Samples	Concentrated Nitric Acid (mL)
FA2	2.5
FA3	7
FA4	10

**Table 4:** The component ratio for synthesizing N-A-S-H gel.

Samples	NaSiO_3_	Al(NO_3_)_3_	NaOH	Initial Si/Al Ratios
N1	200 mL, 0.1 M	200 mL, 0.1 M	10 mL, 10 M	1
N2	200 mL, 0.2 M	200 mL, 0.1 M	10 mL, 10 M	2
N3	200 mL, 0.3 M	200 mL, 0.1 M	10 mL, 10 M	3

**Table 5:** Fitted results of dynamic adsorption experiments for Sr^2+^ with an influent concentration of 100 mg/L under ambient temperature (25 °C) and neutral pH.

Samples	K_TH_	q_max_	K_YN_	Γ	R^2^
N1	0.0070 L/(mg·h)	121.71 mg/g	0.70 h^−1^	5.07 h	0.87
N2	0.0100 L/(mg·h)	111.60 mg/g	1.01 h^−1^	4.60 h	0.91
N3	0.0086 L/(mg·h)	96.28 mg/g	0.86 h^−1^	4.01 h	0.84
FA2	0.0100 L/(mg·h)	115.68 mg/g	1.03 h^−1^	4.68 h	0.84
FA3	0.0059 L/(mg·h)	45.15 mg/g	0.59 h^−1^	1.88 h	0.71

## Data Availability

The original contributions presented in the study are included in the article, further inquiries can be directed to the corresponding author.
